# Effects of albiflorin on oxidative stress and inflammatory responses in rats with acute spinal cord injury

**DOI:** 10.1002/iid3.1015

**Published:** 2023-09-20

**Authors:** Pengfei Fang, Yi Wang, Fengqi Sun, Haisheng Lin, Xindong Zhang

**Affiliations:** ^1^ Department of Orthopedics Hospital of Integrated Traditional Chinese and Western Medicine Baiyin Gansu China; ^2^ Department of Orthopedics Gansu Provincial Hospital of Traditional Chinese Medicine Lanzhou Gansu China; ^3^ Gansu University of Traditional Chinese Medicine Lanzhou Gansu China; ^4^ Department of Orthopedics Second People's Hospital of Baiyin City Baiyin Gansu China

**Keywords:** albiflorin, inflammasome, Nrf2/HO‐1 pathway, oxidative stress, spinal cord injury

## Abstract

**Introduction:**

Oxidative stress and inflammatory responses are often the predominant detrimental factors associated with spinal cord injury (SCI). This study investigates the potential therapeutic effects of albiflorin (AF) on alleviating inflammation and oxidative stress in the rat model with SCI.

**Methods:**

Initially, the behavior of SCI‐induced rats is examined by Basso−Beattie−Bresnahan score and the inclined plane examination. Then, the immunohistochemical staining of inflammasome‐related protein (for instance, NACHT, LRR, and PYD domains‐containing protein 3, NLRP3) is performed in combination with enzyme‐linked immunosorbent assay (ELISA) of corresponding proinflammatory factors to assess the immunomodulatory effects of AF. Further, the markers involved in oxidative stress are examined by ELISA and western blot analysis analyses.

**Results:**

These findings indicated that AF could alleviate motor dysfunction and the loss of neuron cells in SCI‐induced rats. Mechanistically, AF could attenuate the inflammatory responses by reducing oxidative stress and activating nuclear erythroid‐related factor 2 (Nrf2)/heme oxygenase‐1 (HO‐1) pathway in SCI rats. Depleting the antioxidant capacity by inhibiting glutathione biosynthesis could counteract the anti‐inflammatory activity of AF in SCI rats.

**Conclusions:**

Together, our data suggested that AF could serve as a potential therapeutic agent against the aggravation of SCI in rats.

## Graphical abstract

We found that that albiflorin (AF) could reduce the motor dysfunction and loss of neuron cells in spinal cord injury (SCI) rats. Mechanistically, AF can attenuate the inflammatory response by reducing oxidative stress and activating Nrf2/HO‐1 pathway in SCI rats. We have established that AF could be used as a potential agent against SCI in a model organism.

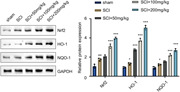

## 1 INTRODUCTION

Acute spinal cord injury (SCI) has emerged as the most common cause of permanent disability, accounting for severe mortality rates in children and adults and posing heavy social and economic burdens.^1^, ^2^ In this context, the typical symptoms of SCI include partial or complete loss of sensory and motor neuronal control functions in limbs and the dysregulation of urinary, respiratory, and neurological functions.^3^, ^4^ In addition, a series of secondary conditions, such as microbial infections, muscle spasms, chronic pain, and depression, ensue after SCI. The surgical correction could stabilize the fractured spinal cord to prevent aggravation.^5^, ^6^

Several important therapeutic interventions based on various drugs and medications have been applied for the primary injury and secondary conditions after SCI.^4^, ^7^ For example, corticosteroids have been used to decrease the swelling in the spinal cord after SCI.^8^ Moreover, suppressing inflammation has emerged as one of the predominant alternatives to protect against the sequelae linked with SCI.^9^ It is increasingly recognized that traditional Chinese medicine (TCM) has shown a promising beneficial effect on alleviating inflammatory damages after acute SCI. Along this line, the concrete mode of action for some TCMs has recently begun to be unveiled.^10^, ^11^, ^12^, ^13^ In an instance, Du Zhong Yao Tong Wan showed restoration function in the SCI‐induced animal model, in which one of the main components, peony (scientifically named *Radix Paeoniae Alba*), could exhibit wound repairing effect.^11^

Further, the biochemical analysis of peony composition resulted in identifying a bioactive compound, albiflorin (AF). Notably, AF has been widely reported in modulating inflammation and oxidative stress in severe pathophysiological conditions.^14^, ^15^, ^16^, ^17^, ^18^, ^19^ In several instances, AF could reportedly mitigate platelet aggregation, possess anti‐coagulation, anti‐thrombosis, and anti‐atherosclerosis effects, promote osteoblast differentiation and femoral fracture healing, as well as improve cognitive functions.^14^, ^15^, ^16^, ^17^, ^18^, ^19^ However, the potential impact of AF treatment on acute SCI and the underlying mechanisms of action remains to be explored.

Since neuroinflammation and oxidative stress can lead to SCI‐induced neurodegeneration^7^, ^8^, ^9^ and AF was reported to protect against inflammation and oxidative stress in different pathophysiological conditions,^14^, ^15^, ^16^, ^17^, ^18^, ^19^ this study is aimed to investigate the potential therapeutic effects of AF on alleviating inflammation and oxidative stress in the SCI‐induced rat model. Initially, we established an SCI rat model and evaluated the treatment effect of AF. Further, AF administration could reduce the extent of motor dysfunction and accelerate the recovery of SCI. After AF treatment, the improved motor function was accompanied by attenuating inflammation and oxidative stress in spinal cord tissues. The beneficial effect of AF was dose‐dependent, and the activation of the nuclear erythroid‐related factor 2 (Nrf2)/heme oxygenase‐1 (HO‐1) pathway might contribute to the therapeutic effect. Together, our data indicated that AF could be used as a potential therapeutic agent against SCI.

## 2 MATERIALS AND METHODS

### 2.1 Preparation of drug extract

The AF solution was prepared as stated below. The AF powder purchased from MedChemExpress Co, Ltd was dissolved in phosphate‐buffered saline (PBS) solution at 20 mg/mL and sterilized by filtration before usage to avoid undesired agglomerates. It should be noted that the purity of AF was above 98%, as indicated in the manufacturer's instructions.

### 2.2 Establishment of SCI rat model

Sprague Dawley (SD) adult male rats (180−250 g; The Jackson Laboratory) were raised in a specific pathogen‐free animal house with providing access to water and food ad libitum. All experimental protocols were approved by the Animal Ethics Committee of Second People's Hospital of Baiyin City and according to the guidelines set out by the Hospital Council on Animal Care (Approval number: 2022004). The SD rats were randomly divided into different experimental groups (Sham, SCI, SCI + different doses of AF), with 10 animals in each treatment group. Initially, the injection site was smeared with 3.6% chloral hydrate. Then, the rats were anesthetized by intraperitoneal injection of 5% isoflurane (Solarbio Life Sciences). Notably, the sham treatment group of animals underwent laminectomy to expose the spinal cord without spinal cord percussion. The SCI‐induced treatment group of animals was subjected to laminectomy, and the exposed spinal cord was placed on the percussion device. To this end, the spinal cord contusion was performed using a 15 g metal rod with a 30 mm free fall for severe injury. Further, the sutures were disinfected with iodophor, and then each rat was injected with 1 mL of penicillin and 2 mL of glucose solution to prevent microbial infection.^13^ After the operation, the SCI‐induced treatment groups were gavaged with different concentrations of AF (50, 100, and 200 mg/kg/day) for further treatment periods (7, 14, 21, and 28 day) before the motor activity assessment. To explore the mechanistic role of the anti‐inflammatory effect attributing to the reduction of oxidative stress, SCI + 200 mg/kg AF group was also administered with l‐Buthionine‐(S, R)‐sulfoximine (BSO) at 5 mg/kg/day (MedChemExpress Co, Ltd). After treatment for predetermined intervals, the rats were killed on Day 28, and the spinal cord tissues were collected for further analysis. A euthanizing chamber was connected to a carbon dioxide (CO_2_) cylinder, and the flow rate was adjusted to displace 40% of the cage volume per minute. The anesthetized rats were validated by placing them into the chamber for 10 min until there was no movement. Later, the death of rats was assured by subsequent cervical dislocation. After laminectomy, the spinal cord was resected, and a part of the tissue was fixed in 4% paraformaldehyde for histological examination. The remaining tissues were snap‐frozen at −80°C and used for biochemical analysis.

### 2.3 Quantification of Basso−Beattie−Bresnahan (BBB) scores

The BBB scores with a 21‐point open‐field locomotor rating scale were used for evaluating the hindlimb movement.^20^ In our experiments, the rats distributed in sham, SCI, SCI + different doses of AF treatment groups were assessed at a 7 day interval after injury for 4 weeks (*n* = 10 per group). It should be noted that the BBB score was determined by the nonexperimental personnel familiar with the scoring criteria. All the rats were observed specifically for three times of 5 min each, and the average score of the three observations was used as the BBB score. The functional tests were conducted in the morning (9:00 a.m. to 11:00 a.m.) of the day, with the BBB score measurement followed by the inclined plane test for the whole experimental period.

### 2.4 Inclined plane test

The inclined plate test was performed to evaluate the recovery of neurological function of rats on 7, 14, 21, and 28 days after SCI procedures.^21^ Briefly, the rats were initially placed on a special inclined plate (50 × 75 cm^2^) with a 1 mm thick grooved rubber surface. Further, the characteristics of maintaining posture, grasping abilities, and the maximum angle of staying on the inclined plate for at least 5 s were measured as their functional score. The functional score was measured thrice for each rat, and the maximum angle of rats staying on the inclined plate was recorded. Notably, the maximum angle of normal rats or the control group staying on the inclined plate should be around 60°.

### 2.5 Nissl staining

The spinal tissues were subjected to Nissl staining after the behavioral tests on Day 28. The collected spinal tissues were snap‐frozen in liquid nitrogen and implanted with the frozen section embedding agent, that is, the optimum cutting temperature (OCT) compound (Leica). The tissues were then sectioned into 5‐μm‐sized thin sections using a microtome. After rehydration the tissue sections were subjected to Nissl staining using the Toluidine Blue dye (Beyotime Biotechnology Co. Ltd) to observe the morphological attributes of the nerve cells. The neuron cells were counted per image field for each group of microscopically observed tissue sections (*n* = 10) using the Image J software (NIH).^22^

### 2.6 Immunohistochemistry (IHC) analysis

Briefly, the collected spinal cord biopsies (size of 6 mm) close to the injury site from the killed rats in different experimental groups were collected on Day 28. After the fixation and embedding with OCT frozen section embedding agent, the tissues were cut into 5‐μm‐sized thin sections using a microtome. After rehydration with alcohol and xylene, the thin sections were subjected to antigen retrieval by heating the tissue section in 1x citrate unmasking solution (Beyotime Biotechnology Co. Ltd) maintained at a subboiling temperature (95°C) for 10 min. Further, the tissue sections were cooled on a bench top at room temperature for 30 min and then incubated in 3% hydrogen peroxide (H_2_O_2_) for 10 min. After rinsing in PBS for 10 min, nonspecific binding sites were blocked by the blocking solution (5% normal goat serum) for 10 min at 25°C. Further, all the samples were incubated with anti‐NACHT, LRR, and PYD domains‐containing protein 3, (NLRP3) monoclonal antibody (1:100 dilution, rabbit, Creative Diagnosis; DCABH‐3606) for 60 min at 25°C. Subsequently, anti‐rabbit immunoglobulin (Ig)G conjugated to fluorescein isothiocyanate (FITC) secondary antibody (1:1000 dilution, ab6717; Abcam) was used to label the sections at room temperature for 60 min. After washing with PBS for 10 min, the sections were counter‐stained with 10 μM 4′,6‐diamidino‐2‐phenylindole (DAPI; Beyotime Biotechnology Co. Ltd) for 10 min. Finally, the incubated tissue sections were washed and observed under a Zeiss fluorescence microscope. The quantitative enumerations of the images were analyzed using Image J software.^23^

### 2.7 Enzyme‐linked immunosorbent assay (ELISA)

The resultant inflammatory mediators, such as interleukin (IL)‐1β, tumor necrosis factor (TNF)‐α, and IL‐6 levels, from the spinal cord tissues in different treatment groups on Day 28 were measured with commercially available ELISA kits (R&D Systems) according to the manufacturer's instructions. Briefly, 100 mg of tissues were ground and lysed using the tissue lysis buffer (1 mL) on ice for 15 min. After centrifugation at 12,000*g* at 4°C for 15 min, the supernatant was collected for ELISA analysis. Initially, 100 μL of the collected supernatant was added to each well in a capture‐antibody‐coated plate for 2 h incubation at ambient temperature. After giving a PBS wash, 50 μL of biotin‐labeled detection antibody was added in each well and incubated for 60 min, followed by the labeling with 50 μL of streptavidin‐horseradish peroxidase (HRP). Further, the signal development was performed by applying 100 μL of chemiluminescent detection reagents for 10 min. Finally, the optical density of samples and standards was measured at 450 nm using a microplate reader. Notably, the concentration of each cytokine was determined based on the linear regression of the standard samples in the kit.

### 2.8 Determination of oxidative stress indicators

The relative levels of various oxidative indicators, such as superoxide dismutase (SOD) and glutathione peroxidase (GSH‐Px) activities, as well as intracellular malondialdehyde (MDA) levels, were detected in the spinal cord tissues collected on Day 28 from different experimental groups. The relative intracellular concentration or activity of each molecule was calculated based on the linear regression of the standards using corresponding biochemical kits (Jiancheng Bioengineering Institute) following the manufacturer's instructions.

### 2.9 Reactive oxygen species (ROS) assay

The intracellular redox status was determined by measuring ROS concentration using the ROS detection kit (R0105; Jiancheng Bioengineering Institute). The spinal tissue samples were initially incubated with 10 μM of 2′,7′‐dichlorodihydrofluorescin diacetate for 45 min at 37°C in the dark. Further, the fluorescence intensity was assessed using a fluorescence spectrophotometer at an oscillation frequency wavelength (Ex/Em) of 485/530 nm. It should be noted that each experiment was conducted in triplicate.

### 2.10 Western blot (WB) analysis

The WB analysis was performed to determine protein expression in the spinal tissues from different treatment groups collected on Day 28. Briefly, 100 mg of spinal cord tissues was initially lysed in 0.5 mL (radioimmunoprecipitation assay, lysis buffer; Beyotime Biotechnology Co. Ltd) on ice for 15 min. Then, the samples were centrifuged at 12,000*g* at 4°C for 15 min. The resultant supernatant was collected, and the precise concentration of protein amount was detected using the bicinchoninic acid protein assay kit (Beyotime Biotechnology Co. Ltd). Further, equal amounts of protein (10 μg) were loaded in each well and separated by 12% sodium dodecyl sulfate‐polyacrylamide gel electrophoresis (SDS‐PAGE). Further, the protein gels were transferred to a nitrocellulose membrane, which was then blocked with a 5% nonfat milk dissolved in a mixture of tris‐buffered saline and tween 20 (TBST) buffer (20 mM Tris, pH 7.5, 150 mM NaCl, and 0.1% Tween 20) for 1 h. Further, the membrane was incubated with primary antibody against glyceraldehyde‐3‐phosphate dehydrogenase (GAPDH, 1:2000; ab191598; Abcam) and custom‐made antibodies against Nrf2, HO‐1, and nicotinamide adenine dinucleotide phosphate quinone dehydrogenase 1 (NQO‐1, 1:1000; Abmart) at 4°C for 8 h. The membranes were washed thrice using TBST buffer and incubated with a second antibody conjugated with HRP (Abcam) for 1 h at 25°C. Further, the signal development was performed using a 3,3′ diaminobenzidine (DAB) HRP substrate (Color Development Kit; Beyotime). Finally, the protein bands were detected using the gel visualization system Alpha Innotech (Tanon‐5200Multi; Tanon). The protein levels were normalized to GAPDH and quantified by the Tanon imaging software (Tanon).

### 2.11 Statistical analysis

Graphpad Prism software version 7 (GraphPad Software) was used for all statistical analyses. Measured data were expressed as mean ± standard deviation. Comparisons among multiple groups were performed by one‐way analysis of variance (ANOVA) with Tukey's post hoc test for pairwise comparison. Data at multiple time points were analyzed by two‐way ANOVA. The difference with *p* < .05 was considered statistically significant.

## 3 RESULTS

### 3.1 AF improves motor function after acute SCI in a rat model

**Figure 1:**
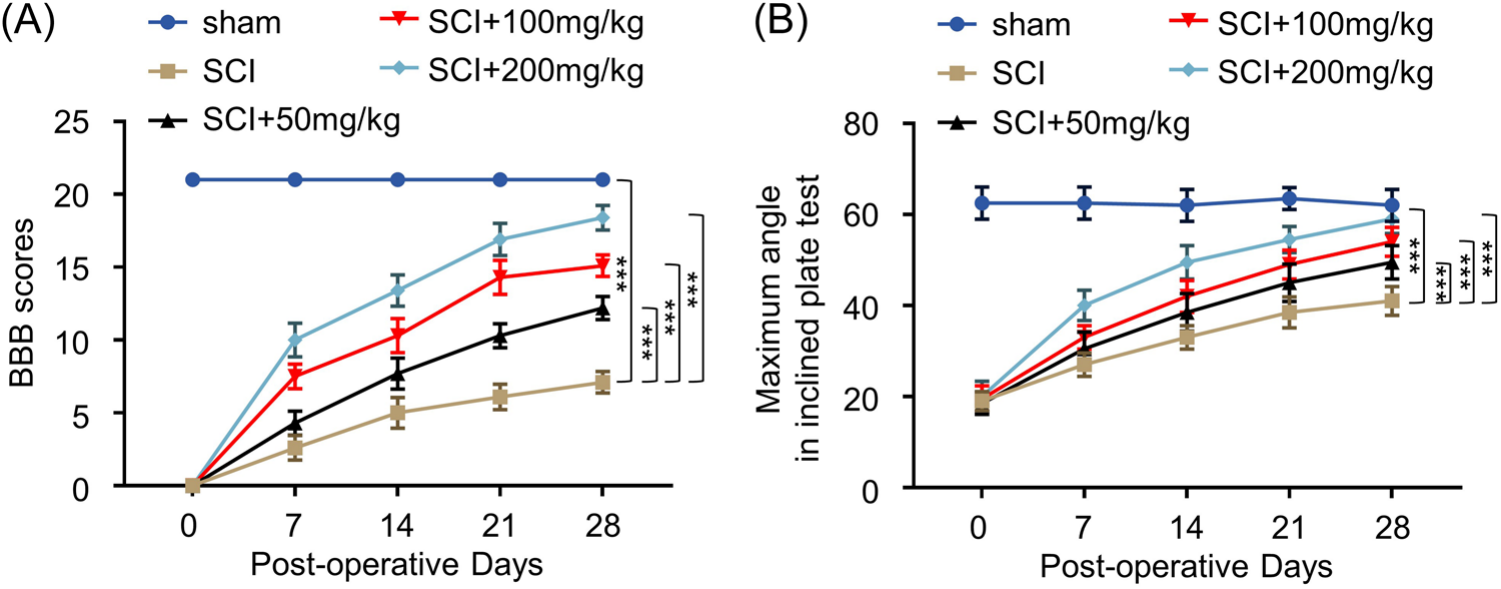
AF treatment improves motor function after acute SCI in rat model. (A) BBB score assessment and (B) inclined plane test were performed to evaluate the motor function in different groups of rats. Data were recorded from four different time points (7, 14, 21, and 28 days) in sham, SCI, and SCI + AF treatment groups (50, 100, and 200 mg/kg/day). Results were expressed as mean ± SD (*n* = 10 rats in each group). ***p < .001; two‐way ANOVA. AF, albiflorin; ANOVA, analysis of variance; BBB, Basso−Beattie−Bresnahan; SCI, spinal cord injury.

This study aims to hypothesize the beneficial effect of AF on alleviating neuronal damage after acute SCI. To explore this study, we established an acute SCI rat model and examined the therapeutic effect of AF by administering different doses of AF (50, 100, and 200 mg/kg/day) for 4 weeks. Further, the motor function was assessed in other treatment groups of rats weekly by BBB 21‐point open‐field locomotor rating scale.^20^ Accordingly, it was observed that AF administration alleviated motor dysfunction caused by acute SCI in a dose‐dependent manner (Figure 1A). Specifically, the motor function tests revealed a seriously disrupted BBB score after SCI induction compared to the sham group. During a 4‐week assessment, the SCI + AF treatment group showed a gradual increase in the BBB score rating. However, the AF group of rats indicated a relatively higher BBB score than the SCI treatment group at each time point. Remarkably, the BBB score of rats in the highest dose of the AF treatment group (200 mg/kg/day) on Day 28 was comparable to that of the sham treatment group. To further corroborate the observations in the BBB rating scale, we applied the inclined plane test to examine the motor balancing ability of the rats in different treatment groups. Similar to the BBB score, the maximum angle of rats staying on the inclined plane gradually increased after SCI induction in both the SCI and SCI + AF treatment groups. Moreover, the AF treatment groups displayed an enhanced plane climbing ability (Figure 1B). The inclined plane test showed an increase in the climbing ability of rats with the highest AF dose of 200 mg/kg/day by threefolds (from a 20 to a 58° angle) after 4‐week treatment. Together, these data suggested that AF treatment could substantially alleviate motor dysfunction and improve the balancing ability of rats after acute SCI induction.

### 3.2 AF reduces the loss of neurons after acute SCI

**Figure 2:**
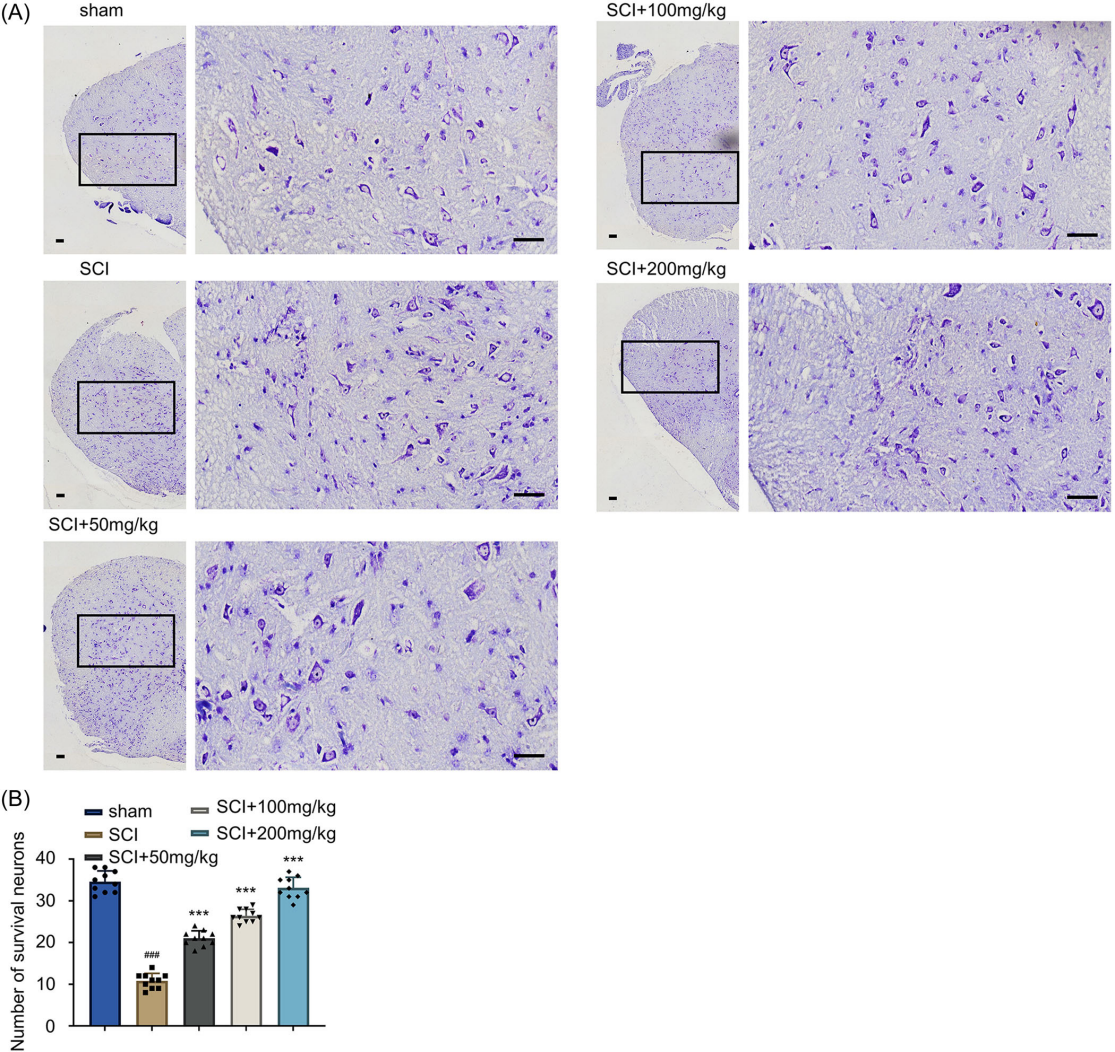
AF treatment reduces the loss of neurons after acute SCI. (A) Nissl staining of the spinal tissue sections collected on Day 28 in sham, SCI, and SCI + AF treatment groups (50, 100, and 200 mg/kg/day). Dark blue indicates the nuclei of neuron cells. The zoomed square region shows neuronal degeneration. Scale Bar = 40 μm (×200 magnification). (B) Statistical analysis of number of survival neurons in the tissue sections of different groups of rats. Results were expressed as mean ± SD (*n* = 10 in each group). ***p < .001 versus SCI group; ^###^ *p* < .001 versus sham group; one‐way ANOVA. AF, albiflorin; ANOVA, analysis of variance; SCI, spinal cord injury.

Since motor function is closely related to neuronal activity, we further validated the role of AF treatment in improving the survival of neurons in the spinal cord after SCI. To explore this aspect, Nissl staining was performed to examine the morphological and pathological characteristics of spinal cord tissue sections of rats in different experimental groups collected on Day 28. The tissue sections of the SCI‐induced treatment group showed tissue cavitation and cell degeneration (Figure 2A). Moreover, the AF treatment group showed a dose‐dependent protective effect against tissue degeneration. The quantification of neuron number in the tissue sections suggested that the SCI induction significantly caused severe neuron loss in the spinal cord compared to the sham group (Figure 2B, *p* < .001). Furthermore, the AF treatment group showed higher neuron cell counts after SCI induction in a dose‐dependent manner compared to the SCI group (Figure 2B, *p* < .001). These data demonstrated the neuroprotective of AF treatment against acute SCI.

### 3.3 AF attenuates the inflammatory response induced by SCI

**Figure 3:**
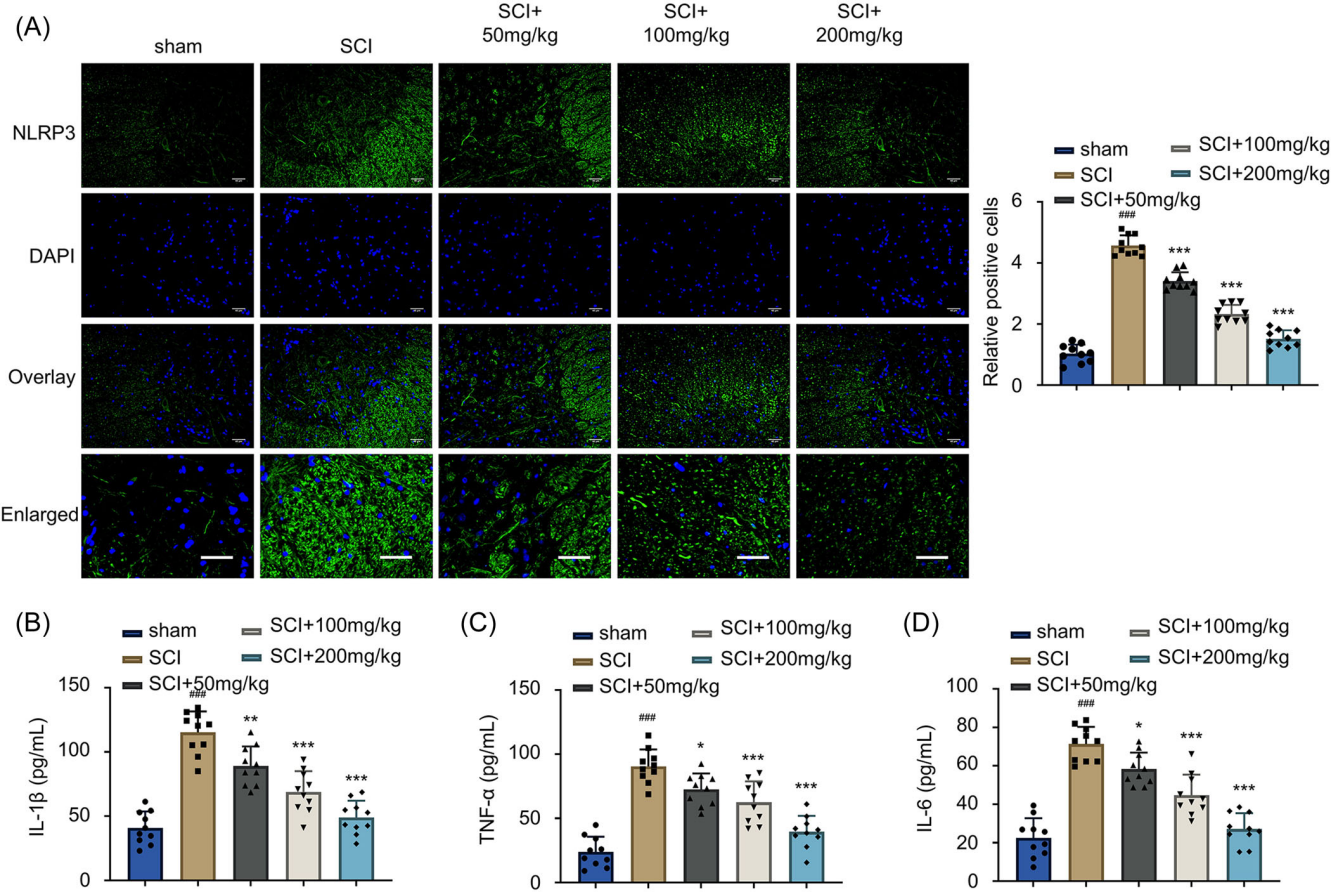
AF administration attenuates the inflammatory response induced by SCI. (A) IHC staining of NLRP3 in the spinal tissue sections collected on Day 28 in sham, SCI, and SCI + AF treatment groups (50, 100, and 200 mg/kg/day). Nulei were stained with 4,6‐diamidino‐2‐phenylindole (DAPI). The fluorescence intensity of NLRP3 was compared between sham, SCI, and SCI + AF treatment groups. Scale Bar = 40 μm (×200 magnification). Results were expressed as mean ± SD (*n* = 10 in each group). ***p < .001 versus SCI group; ^###^ *p* < .001 versus sham group. (B) IL‐1β, (C) TNF‐α, (D) IL‐6 levels in the spinal tissues were analyzed by ELISA in the sham, SCI, and SCI + AF treatment groups. Results were expressed as mean ± SD (*n* = 10 in each group). *p < .05, **p < .01, and ***p < .001 versus SCI group; ^###^ *p* < .001 versus sham group; one‐way ANOVA. AF, albiflorin; ANOVA, analysis of variance; ELISA, enzyme‐linked immunosorbent assay; IHC, immunohistochemical; SCI, spinal cord injury; TNF, tumor necrosis factor.

Notably, neuroinflammation plays a key role in SCI‐induced neurodegeneration.^9^ Accordingly, we further examined the activity of NLRP3 inflammasome and detected the intracellular expression levels of proinflammatory cytokines in the spinal cord tissues of different treatment groups. The IHC staining showed that the NLRP3 inflammasome activity was dramatically increased upon SCI induction compared to the sham group (Figure 3A, *p* < .001). Moreover, the AF treatment group of rats showed suppressed NLRP3 inflammasome activity in a dose‐dependent manner after SCI induction than the SCI group (Figure 3A, *p* < .001). The ELISA measurements of the relative levels of IL‐1β, TNF‐α, and IL‐6 in the spinal cord tissues showed that the SCI‐induction group showed significantly higher levels than the sham group (Figure 3B−D, *p* < .001). Similarly, the AF administration suppressed the production of IL‐1β, TNF‐α, and IL‐6 levels in a dose‐dependent manner compared to the SCI‐induction group (Figure 3B−D, *p* < .05, *p* < .01, or *p* < .001). Collectively, AF treatment could efficiently reduce the inflammatory response triggered by acute SCI in rats.

### 3.4 AF reduces oxidative stress in rats with acute SCI

**Figure 4:**
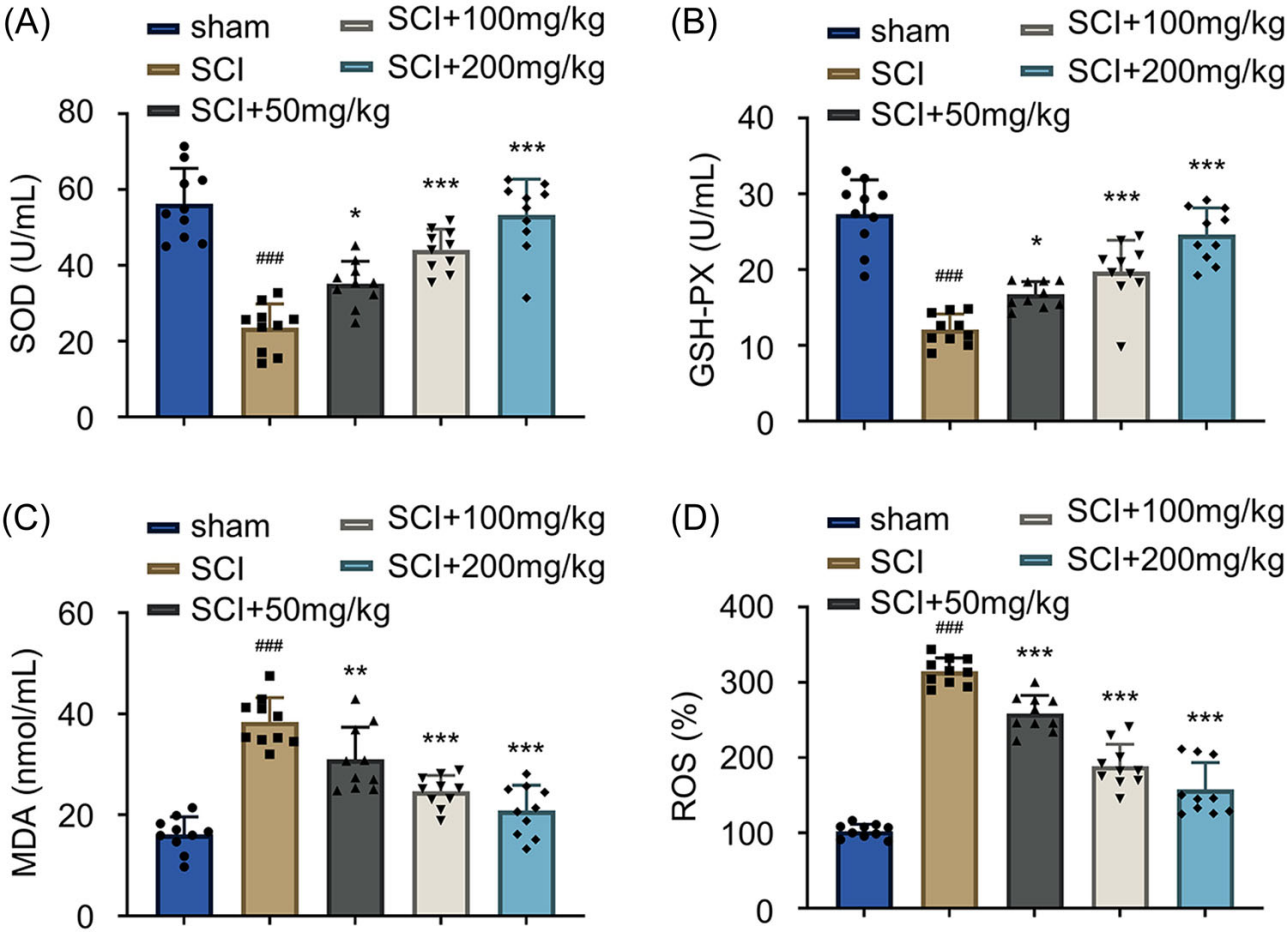
AF treatment reduces oxidative stress in rats with acute SCI. (A) SOD, (B) GSH‐Px, (C) MDA, and (D) ROS levels were detected in the spinal tissues collected on Day 28 in sham, SCI, and SCI + AF treatment groups (50, 100, and 200 mg/kg/day). Results were expressed as mean ± SD (*n* = 10 in each group). *p < .05, **p < .01, and ***p < .001 versus SCI group; ^###^ *p* < .001 versus sham group; one‐way ANOVA. AF, albiflorin; ANOVA, analysis of variance; GSH‐Px, glutathione peroxidase; MDA, malondialdehyde; ROS, reactive oxygen species; SCI, spinal cord injury; SOD, superoxide dismutase.

In addition, oxidative stress plays an important role in the SCI pathogenesis.^23^, ^24^ To explore the role of AF, we investigated whether AF treatment could attenuate the SCI‐induced oxidative burden. The SOD levels, an enzyme for the detoxification of superoxide, were reduced in the spinal cord tissues of SCI rats compared to the sham treatment group (Figure 4A, *p* < .001). In contrast, the AF treatment showed significantly increased SOD levels with an increase in the AF dose (Figure 4A, *p* < .05 or *p* < .001). In addition, we examined the activity of another antioxidant enzyme, that is, the selenium‐dependent GSH‐Px.^25^ Similarly, an AF‐dependent upregulation of GSH‐Px activity was observed under the SCI conditions (Figure 4B, *p* < .05 or *p* < .001), indicating that AF treatment could enhance the ROS detoxification capacity in the spinal cord tissues. Contrarily, the MDA levels, a marker of oxidative stress involved in the lipid peroxidation,^26^ were significantly increased in the spinal tissues of the SCI group compared to the sham group (Figure 4C, *p* < .001). In contrast, AF treatment showed dose‐dependent effects in reducing MDA levels over the SCI treatment group (Figure 4C, *p* < .01 or *p* < .001). In addition, the ROS levels were detected in each experimental group. The SCI induction significantly elevated the ROS levels in the spinal cord tissues. Contrarily, AF treatment showed dose‐dependent effects in reducing the ROS levels in the SCI group compared to the SCI group (Figure 4D, *p* < .001). Together, these findings suggested that AF treatment substantially alleviated oxidative stress by augmenting the ROS detoxification ability.

### 3.5 AF activates Nrf2/HO‐1 pathway

**Figure 5:**
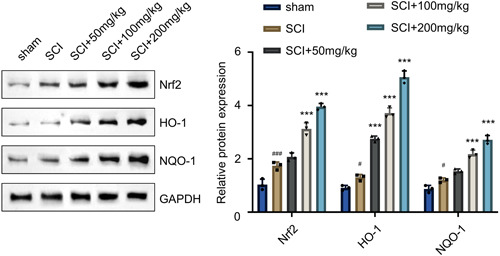
AF treatment activates Nrf2/HO‐1 pathway. Western blot analysis of Nrf2, HO‐1, and NQO‐1 in the spinal tissues collected on Day 28 in sham, SCI, and SCI + AF treatment groups (50, 100, and 200 mg/kg/day). GAPDH was used as a housekeeping control. Results were expressed as mean ± SD (*n* = 3 samples analyzed in each group). *p < .05, ***p < .001 versus SCI group; ^#^ *p* < .05, ^###^ *p* < .001 versus sham group; one‐way ANOVA. AF, albiflorin; ANOVA, analysis of variance; SCI, spinal cord injury.

Furthermore, the mechanistic effect of AF was explored. Since AF could reduce oxidative stress in SCI rats, we wondered whether AF could regulate the canonical antioxidant response signaling mediated by Nrf2/HO‐1 pathway.^27^, ^28^, ^29^ In this vein, several reports have also implicated the neuroprotective mechanism of several CTMs. To demonstrate this aspect, the WB analysis was performed to examine the expression levels of Nrf2, HO‐1, and NQO‐1 in the spinal cord tissues of different treatment groups. It was observed that, in contrast to the sham group, the protein levels of Nrf2, HO‐1, and NQO‐1 showed an increasing trend in the SCI‐induction treatment group (Figure 5, *p* < .05 or *p* < .001). Moreover, AF treatment enhanced the expression of these notified proteins in a dose‐dependent manner in the spinal cord tissues of the rats compared to the SCI group (Figure 5, *p* < .05 or *p* < .001). Together, these data suggested that AF could activate Nrf2/HO‐1 pathway, thus alleviating the oxidative stress after SCI induction in rats.

### 3.6 The attenuation of oxidative stress accounts for the anti‐inflammatory activity of AF treatment in SCI rats

**Figure 6:**
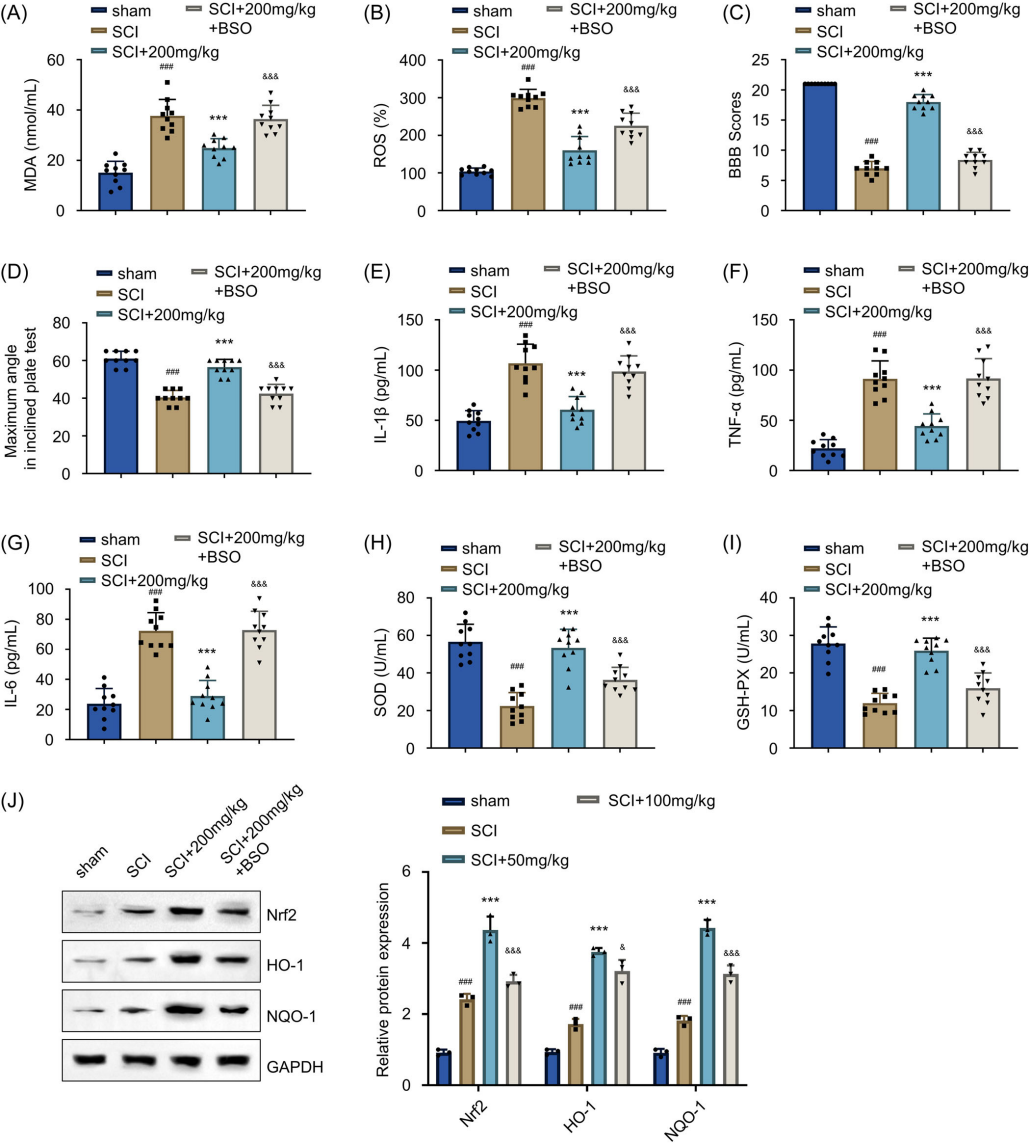
The attenuation of oxidative stress accounts for the anti‐inflammatory activity of AF treatment in SCI rats. (A) MDA and (B) ROS levels in the spinal cord tissues collected on Day 28 in sham, SCI, and SCI + AF (200 mg/kg/day) and SCI + AF + BSO (5 mg/kg/day) treatment groups. The graphs present (C) BBB score assessment and (D) inclined plane test to evaluate the motor function in different groups of rats. Data are recorded from four other time points (7, 14, 21, and 28 days). The ELISA data present (E) IL‐1β, (F) TNF‐α, and (G) IL‐6 levels in the spinal cord tissues collected on Day 28 in each experimental group. The images present (H) SOD and (I) GSH‐Px levels detected in the spinal tissues collected on Day 28 in each experimental group. (J) The WB analysis shows the Nrf2, HO‐1, and NQO‐1 proteins in the spinal tissues collected on Day 28 in each experimental group. Results were expressed as mean ± SD (*n* = 10 in each group). For WB analysis, *n* = 3 samples were analyzed in each experimental group. ***p < .001 versus SCI group; ^###^ *p* < .001 versus sham group; ^&&&^ *p* < .001 versus SCI group + 200 mg/kg/day AF group; one‐way ANOVA. AF, albiflorin; ANOVA, analysis of variance; BBB, Basso−Beattie−Bresnahan; ELISA, enzyme‐linked immunosorbent assay; GSH‐Px, glutathione peroxidase; MDA, malondialdehyde; ROS, reactive oxygen species; SCI, spinal cord injury; SOD, superoxide dismutase; TNF, tumor necrosis factor; WB, western blot analysis.

Since we uncovered that AF could reduce the inflammatory response and mitigate oxidative stress in the SCI model, we hypothesized that the attenuation of oxidative burden could be responsible for the anti‐inflammatory effect. Therefore, we included another group of SCI rats, which were administered with 200 mg/kg/day AF along with BSO, an inhibitor of antioxidant glutathione biosynthesis, at 5 mg/kg/day. The detected MDA and ROS levels in the spinal cord tissues showed that the BSO administration substantially promoted oxidative burden upon AF treatment compared to the SCI + AF treatment group (Figure 6A,B, *p* < .001). Further, the motor function analysis by BBB score and the inclined plane test showed that the BSO administration abrogated the rescue effect of AF treatment on motor dysfunction (Figure 6C,D). In addition, ELISA measurement of IL‐1β, TNF‐α, and IL‐6 in the spinal cord tissues exhibited that the suppressive effects of AF treatment on these inflammatory cytokines were substantially abolished in the presence of BSO (Figure 6E−G, *p* < .001). Moreover, the effects of AF treatment on promoting the SOD expression and increasing the GSH‐Px levels were suppressed by BSO administration (Figure 6H,I, *p* < .001 compared to the SCI + AF treatment group). Consistently, the AF‐dependent upregulation of different antioxidant proteins (Nrf2, HO‐1, and NQO‐1) was suppressed by BSO (Figure 6J, *p* < .001 compared to the SCI + AF treatment group). Together, these findings demonstrated that impairing the antioxidant capacity by glutathione biosynthesis inhibition could substantially counteract the anti‐inflammatory effects of AF in SCI rats. Considering these aspects, these data indicated a pivotal role of oxidative stress attenuation in the neuroprotective effect of AF treatment.

## 4 DISCUSSION

This study revealed that the bioactive compound AF extracted from a peony (*R. Paeoniae Alba*) could efficiently improve motor function and reduce the loss of neuron cells in SCI rats. In addition, AF could reduce inflammatory responses by significantly attenuating oxidative stress and activating the Nrf2/HO‐1 pathway in SCI rats. Overall, these findings highlighted that AF could be used as a potential agent against SCI in model organisms.

TCM is a valuable resource of bioactive components, exhibiting anti‐inflammatory effects to protect against diseases, including SCI.^10^, ^11^, ^12^, ^13^, ^30^, ^31^ For instance, paeoniflorin, an extract from peony, has been reported to possess various biological activities in modulating the MAPK pathway to recover chronic constriction injury in rats.^32^ Owing to these aspects, researchers have garnered enormous attention in raising the possibility that compounds similar to paeoniflorin, such as AF, could act as a protective agent for treating acute SCI. Similarly, other natural extracts have also been revealed to benefit neuropathic injuries. Among various traditional medicines available, curcumin has been shown to curtail inflammatory damage by inhibiting nuclear factor‐kappa B (NF‐κB) signaling, a critical transcription factor involved in the inflammatory activation of the immune response, apoptosis, and carcinogenesis.^29^

In this context, the underlying mechanisms of SCI have been studied extensively. It has been increasingly recognized that secondary injuries are linked with cellular, molecular, and biochemical changes in neurons following SCI induction.^33^ Along this line, the predominant consequences of SCI include the production of free radicals and the oxidation of lipids and proteins. In addition to the oxidative damage, neuroinflammation mediated by the activation of immune cells, such as macrophages or microglia, is another key detrimental factor contributing to neurodegeneration.^34^ In an instance, it was demonstrated that activating cytoplasmic NLRP3 inflammasome complex could trigger neuronal cell death through pyroptosis, aggravating inflammation.^35^ In our study, we demonstrated that the protective effect of AF in SCI rats could be abolished by applying a glutathione synthesis inhibitor, BSO. BSO administration substantially aggravated oxidative burden and abrogated the anti‐inflammatory activity of AF treatment. Thus, we concluded that the attenuation of oxidative stress could account for the anti‐inflammatory activity of AF treatment in SCI rats.

In the past decade, AF has been extensively studied to explore its versatile roles in treating obesity, memory deficit, cognitive function, and inflammation.^14^, ^15^, ^19^, ^36^ In this study, we expanded its application potential in treating SCI. Further, we showed that Nrf2/HO‐1 pathway was mildly activated in SCI rats, which might antagonize the increased oxidative burden. Moreover, AF treatment strongly promoted the activation of the Nrf2/HO‐1 pathway in a dose‐dependent manner. Several reports demonstrated that the Nrf2/HO‐1 pathway possessed a key antioxidant response component in eukaryotic cells.^37^ Moreover, the Nrf2‐dependent gene program and its target protein HO‐1 exerted beneficial effects in development and pathogenic conditions by protecting against oxidative injury, regulating apoptosis, and modulation of inflammation.^38^, ^39^ The perturbation of proper HO‐1 expression or activity could be associated with the pathogenesis of age‐related disorders, including neurodegeneration and neurovascular dysfunction.^40^ The activation of the Nrf2/HO‐1 pathway has been reported to inhibit the inflammatory activation of macrophages.^41^ Along this line, several natural compounds, such as curcumin, isoliquiritigenin, and anthocyanins, have been reported to activate Nrf2/HO‐1 pathway to mitigate the inflammatory and oxidative damages in the animal models of SCI, pancreatitis, and Alzheimer's disease.^29^, ^42^, ^43^ Therefore, the anti‐inflammatory and antioxidant effect of AF treatment might be attributable to the activation of the Nrf2/HO‐1 pathway.

Despite the findings guiding the effect of AF and its underlying mechanism, several questions still need to be further addressed. First, we only included three samples for WB analysis in each animal group. Future work should contain more biological samples given the variation in individual animal. Second, the mechanism by which AF activates the Nrf2/HO‐1 pathway requires further investigation. Third, the specific effects of AF treatment on different populations of immune cells in the SCI model need to be explored. Lastly, evaluating the combinatory effect of AF and other therapeutic compounds in the SCI model is necessary to formulate an optimal intervention strategy for SCI treatment.

## 5 CONCLUSIONS

In summary, our study has demonstrated the potential therapeutic effect of AF on alleviating inflammation and oxidative stress in the rat model of SCI. In addition, AF treatment accelerated the recovery of motor function after SCI induction. Mechanistically, AF treatment suppressed the inflammation by reducing oxidative stress and activating the Nrf2/HO‐1 pathway in SCI rats. Thus, these data showed the therapeutic potential of AF to mitigate neurodegeneration after SCI.

## AUTHOR CONTRIBUTIONS

*Designed and performed the experiments*: Xindong Zhang, Pengfei Fang, and Yi Wang. *Statistical analysis*: Pengfei Fang, Yi Wang, Fengqi Sun, and Haisheng Lin. *Writing—original draft*: All authors. All authors read and approved the final manuscript.

## CONFLICT OF INTEREST STATEMENT

The authors declare no conflict of interest.

## ETHICS STATEMENT

All experimental protocols were fully complied with and approved by the Animal Ethics Committee of Second People's Hospital of Baiyin City and according to the guidelines set out by the Hospital Council on Animal Care (Approval number: 2022004).

## Data Availability

The data sets used and analyzed during the current study are available from the corresponding author via email request.
